# Mental health challenges of recent immigrants in precarious work environments — a qualitative study

**DOI:** 10.3389/fpsyt.2024.1428276

**Published:** 2024-09-03

**Authors:** Janki Shankar, Shu-Ping Chen, Daniel W. L. Lai, Shawn Joseph, Rhea Narayanan, Zabin Suleman, H M Ashraf Ali, Priyadarshini Kharat

**Affiliations:** ^1^ Faculty of Social Work, University of Calgary, Edmonton, AB, Canada; ^2^ Faculty of Rehabilitation Medicine, University of Alberta, Edmonton, AB, Canada; ^3^ Dean and Chair Professor, Faculty of Arts and Social Sciences, Hong Kong Baptist University, Hong Kong, Hong Kong SAR, China; ^4^ Horace Greeley High School, Chappaqua, New York, United States; ^5^ Department of Anthropology, Economics and Political Science, MacEwan University, Edmonton, AB, Canada

**Keywords:** recent immigrants, precarious employment, mental health, well-being, overqualified

## Abstract

**Introduction:**

Recent immigrants from racialized minority backgrounds and those who are not proficient in the local language are some of the most vulnerable members of society. Despite having postsecondary educational qualifications and permanent residency status, many are engaged in precarious employment. There is a scarcity of research that has explicitly focused on the work experiences and mental health challenges faced by these immigrants.

**Methods:**

Using a grounded theory approach and semi-structured face-to-face interviews, this study examined the work experiences and mental health challenges of 42 recent immigrant employees from two cities in Canada who were working in various industries and engaged in precarious employment.

**Findings:**

Eighty-one percent of the employee participants were overqualified for their jobs. Findings highlighted several ongoing mental problems that participants experienced, stemming from challenging physical and psychological workplace conditions, negative mindsets associated with their recent immigrant status, and other contextual factors and barriers. However, various coping strategies, both constructive and unconstructive, were used to address this mental distress.

**Discussion:**

The study proposes a multidimensional approach to address workplace conditions to promote good mental health for these employees. This includes preventative programs for raising awareness among employers about the importance of recent immigrant employees’ mental health and well-being and policy and legislation changes to ensure the employer’s commitment to creating a safe and culturally friendly workplace. The approach also recommends that recent immigrant employees receive occupational health and safety training, learn about Canadian workplace norms and culture, and have access to professional healthcare services.

## 1 Introduction

Many immigrants, driven by the need to enhance their quality of life, safety, and social mobility, uproot themselves and their families to move to a new place. Though not without challenges, their journey is a testament to their resilience and determination (1). For most immigrants, meaningful and gainful employment is critical for their adaptation, integration, and physical and mental health. However, despite having post-secondary qualifications or professional/trade expertise from their home country, many recent immigrants (those in the host country for less than ten years (2) often find themselves in precarious employment (PE) conditions below their educational levels, skills, and experience.

### 1.1 The pervasive nature of precarious employment

There is no universally accepted definition of PE (3). Still, a systematic review of definitions and operationalizations from quantitative and qualitative studies highlights that PE is a multidimensional construct that includes three dimensions: (a) job insecurity, (b) income inadequacy, and (c) lack of employee rights and protection. The last dimension refers to the lack of unionization among employees, lack of social benefits like employer-funded pension, medical benefits, or leave, and lack of workplace rights like protection against unfair dismissal, protection from authoritarian treatment, discrimination, or harassment (3–5). Some conceptualizations of PE also include poor workplace social support, lack of access to training and development, low work status, and high exposure to occupational health hazards (6). PE has been increasing globally since the 1980s and has been attributed to factors such as the weakening of labor unions, the digitalization of the workforce, the rise of the discourse of individualism and personal responsibility, and the takeover of the economy by financial sectors and elites who influence economic policy and outcomes (4). These structural shifts have emboldened employers to relax employment standards and increase the number of low-paid contractual workers with poor access to collective representation (4). Although precarious employment has always been most prevalent among immigrants, racialized groups, and women, it is now found in all income levels and demographic groups in current employment systems. PE encompasses many jobs ranging from semi-skilled ones requiring only on-the-job training to skilled work that requires high qualifications (7). According to Statistics Canada, 2018 (8), over 30% of Canadians in Canada work under temporary, seasonal, or contract employment. Research shows PE can adversely affect employees’ health and well-being (3, 7). The risks associated with PE include musculoskeletal and cardiovascular diseases (9, 10) and mental health conditions like stress and anxiety, depression, sleep disturbances, and burnout (11, 12).

### 1.2 Immigrants and precarious employment

Several international studies show a high prevalence of mental disorders such as depression, anxiety, and substance abuse among immigrant employees. These issues are attributed to precarious employment, workplace psychological stressors, poor working conditions, and abuse, as well as various personal, social, and environmental factors (13, 14). The International Labor Organization (ILO)reports that there are approximately 164 million immigrant employees across the globe, and about two-thirds of them live in high-income countries like Canada, Australia, and the United States, where they are an essential labor resource performing semiskilled jobs (15). These immigrants comprise the host country’s permanent residents (PR), temporary foreign workers (TFWs), international students with work permits, and undocumented immigrants without legal immigration status and work permits. In Canada, PR status grants the individual the right to live and work permanently in Canada, study, and access most social benefits, including healthcare and social services, similar to Canadian citizens. TFWs and international students are bound by specific conditions (8, 16), such as the length of their stay in Canada and the nature of work or study they undertake, and have restrictions on accessing certain social benefits. However, TFWs have the same rights and protections as PRs and are eligible for provincial/territorial health care plans. While selecting newcomers for permanent resident status, countries like Australia and Canada attract relatively young, professionally qualified, and skilled immigrants with language proficiency, job offers, or in-demand skills (17). Yet, when these newcomers land in the host country, many get funneled into precarious labor market conditions and undertake jobs for which they are overqualified (18). This experience can affect many skilled immigrants’ physical and mental health (19). The critical reasons for their loss of professional status include a lack of recognition of foreign credentials, delayed assessment by provincial regulatory bodies, and a lack of Canadian employment experience (20). While some move into better jobs over time, many qualified immigrants continue in such employment for extended periods (20). Additionally, language and communication barriers, lack of social support and professional networks, and poor understanding of health care and labor laws expose many of these individuals to employer prejudice, racism, harassment, and exploitation (21, 22). Immigrant employees who do not have PR status may experience additional challenges, like being asked to perform jobs that are not in their contracts under threat of deportation (22). These challenges are exacerbated by failures in employment laws and regulations and other social and cultural issues related to absorbing new immigrants. For instance, employment laws may not provide adequate protection for immigrant workers, and due to sociocultural barriers such as discrimination, they are left vulnerable to exploitation. All these factors increase the risk of immigrant employees developing mental health problems (22).

Although precarious employment can adversely affect the health of all employees, its health effects on the general population can differ from those on immigrants, who can vary from non-immigrants in their health status, expression of health problems, access to healthcare services, and health-related risk factors (23, 24). Hence, there is a need for research that focuses explicitly on examining how precarious employment conditions impact the mental health and well-being of immigrant groups, like recent immigrants who are permanent residents and temporary foreign workers (TFWs) (24).

Several quantitative studies (25–27) have shown a positive causal association between precarious employment and poor mental health, but the mechanisms that explain this relationship have not been sufficiently examined in research (28–30). Qualitative research that focuses on employees’ lived experiences can help in understanding how and why precarious employment has negative implications for the mental health of immigrant employees and what coping strategies these individuals may use to manage their challenges.

Given these research gaps, the objective of the current study was to examine the work experiences and associated mental health challenges faced by recent immigrant employees in the context of precarious employment. This study specifically focused on recent immigrants with PR status for two reasons: first, there is relatively little research on the work experiences and mental health challenges faced by this population, and second, this group would seem to be potentially less vulnerable to exploitation by employers compared to TFWs and undocumented workers who do not have PR status. The findings can help countries that receive a high number of qualified, skilled immigrants, like Canada, understand the multifaceted challenges immigrant employees face due to the intersectionality of mental health, working conditions, and recent immigrant status.

### 1.3 Theoretical perspectives: understanding immigrant employees’ experiences from an intersectionality perspective

Recently, some studies have used the intersectionality perspective as a lens to understand the experiences and needs of immigrants in developed countries (31, 32). According to this perspective, multiple factors can shape subjective well-being (33), which refers to people’s appraisal of various domains of their lives, like health, work, family, or feelings, which can be both positive and negative. Positive feelings may include pleasure, while negative emotions may include pain, worry, and anger (34). When applied to recent immigrant employees, the intersectionality perspective helps in understanding how being a member of multiple marginalized groups puts them at risk of being treated unequally, thus making them more vulnerable to negative experiences and exacerbating their mental health risks.

This study also draws on Smith and colleagues’ occupational health and safety (OHS) vulnerability framework (35), which identifies four dimensions that raise employee risk of physical and psychological injury. These are poor awareness of OHS, workplace hazards exposure, poor workplace protections, and a workplace culture that discourages employees from asking questions about safety. This framework can also be applied to recent immigrants who may have limited OHS understanding and training, are likely to engage in precarious employment, and may feel they need more support to voice their safety concerns.

Although the current study draws from multiple perspectives and frameworks as a conceptual base, it does not adopt or test a specific theory or framework.

### 1.4 Research questions

The primary research question was: What are the work experiences of recent immigrant employees with permanent residency status engaged in precarious employment, and how did these contribute to their mental health and well-being? A secondary research question was: What strategies do recent immigrants use to manage their work-related challenges?

### 1.5 Ethical Statement

The current study is part of a larger study titled “Immigrant worker, service provider, and employer perspectives on the occupational safety and work conditions of immigrant workers, and their return-to-work experiences after occupational injury or illness.” Before beginning the data collection for the study, ethical approval was obtained from the Research Ethics Board at the lead author’s university. Each study participant was fully informed about the objectives and procedures of the study before their interview, the risks and benefits of participating, and that their participation was voluntary. All the participants provided written consent for their participation.

This study was conducted in Canada. Most studies on the labor market and work experiences of immigrants in Canada have been conducted in major Canadian cities like Toronto, Vancouver, and Montreal, which are traditionally the preferred destinations for newcomers (36). With a notable shift in the settlement patterns of newcomers to smaller urban centers in recent years (36), there is a need for research on the work experiences of newcomers in these centers with fewer resources. The current study addresses this need. The participants for this study were recruited from Edmonton and Calgary, the two largest cities in Alberta province with a high proportion of immigrants (37).

## 2 Materials and methods

### 2.1 Research design

Given the exploratory nature of this research, a constructivist grounded theory approach (38) was chosen to understand and explain recent immigrant employees’ work experiences and their influence on the mental health and well-being of study participants. This approach positions the researcher as a co-author in the (re)construction, interpretation, and representation of knowledge, experiences, and meanings while retaining participants’ own words and stories in written representations (38).

### 2.2 Conceptualizing mental health and well-being

This study conceptualizes mental health from a psychosocial instead of a clinical perspective. The participants were not asked specific Western medicalized questions about symptoms or diagnosis. Given the personal nature of all psychological experiences and ongoing debates about the utility of diagnostic measures (39) and in keeping with several qualitative studies that do not use standardized measures to assess mental health (40), this study’s assessment of the participant’s mental health and well-being was based on subjective accounts of their psychological states as elicited during the research interviews.

### 2.3 Study sample

A purposive sampling approach was used to recruit participants. To be eligible to take part in the study, the person had to be: (a) from backgrounds that are racialized and non-English speaking, (b) between 18 -60 years, (c) a permanent resident (PR) or Canadian citizen for < 10 years, (d) currently working or has gained Canadian work experience for at least two years as a PR and (e) working in a job that they perceived was unsafe and had a high chance of getting injured. Those excluded from this study were temporary foreign workers and undocumented laborers. Eligible participants were recruited with the help of service providers from immigrant-serving organizations who had advertised the study through their client networks. Some were recruited through the researchers’ community networks.

### 2.4 Data collection

Recruitment and data collection started on August 2, 2017, and were completed on November 28, 2019. The lead investigator(JS) and two trained graduate research assistants (AA, ZS) conducted face-to-face and in-depth interviews using semi-structured interview schedules. Each interview lasted for 1.5 hours and was conducted to understand the participants’ experience of job hazards, the conditions under which they worked, the physical and emotional challenges they experienced, and their awareness of the safety aspects of their jobs (refer to **Appendix**). Further probes, clarifications, and questions were developed in response to participants’ answers. The research team developed the interview schedule in consultation with immigrant service providers. The interviews were held in the principal investigator’s office or a location chosen by the participant. The interviews were conducted in English since participants reported adequate fluency. However, since many participants did not have enough vocabulary to narrate their experiences, the interviewers gave them enough time to complete what they wanted to convey and used simple sentences and examples to explain a question or concept. The interviewers also paraphrased statements to ensure they accurately captured what participants wanted to say. The interviews were audiotaped and transcribed verbatim after obtaining the participants’ written consent. Each participant received an honorarium of $50 for their participation.

### 2.5 Data analysis

Data collection and analysis were done simultaneously. During the interviews, the interviewers made notes of meaningful discussions. During research team meetings, the group discussed biases arising from interviewers ‘positionality and professional beliefs, theoretical orientation, immigration status, social positionality, coding decisions, interview notes, and their emotional responses toward participants.

The data analysis process was characterized by transparency and openness, beginning with loading verbatim transcriptions onto Atlas—ti 7 Software for initial coding and framework development. The grounded theory approach, with its emphasis on iterative data collection and analysis, constant comparison, theoretical sampling, and memo writing, ensured that our findings were deeply rooted in empirical data (38). To further ensure the study’s trustworthiness, two research assistants (SJ, ZS) coded independently and then compared codes to ensure inter-coder reliability. The research team rigorously assessed internal validity, external validity, reliability, and objectivity based on credibility, transferability, dependability, and confirmability (41–43). The resulting codes were organized under broad thematic headings related to the study objectives. Despite achieving theoretical saturation (41, 42) after analyzing thirty transcripts, we chose to analyze all 42 interview transcripts. The entire research process and analysis procedures were meticulously documented, and hard copies of the transcripts were maintained.

## 3 Results

### 3.1 Demographic characteristics of study participants

**Table 1**: Demographic characteristics of participants.

**Table 1:** Sociodemographic data of participants.

Attributes	Injured	Non Injured	Injured but did not report
Gender			
Men	12	10	1
Women	12	5	2
	(9 not working)		
Age			
18-25	1		
26-35	6	2	
36-45	8	5	2
46-55	9	8	1
Education			
High school	5	3	
Undergraduate degree	10	4	2
Master’s degree	8	7	1
PhD/MD	1		
Overseas trade certificate		1	
Number of years in Canada			
1-2 years	3	4	
3-5 years	12	6	2
6-< 10 years	9	5	1
Region of Origin			
Asia	11	5	2
Africa	5	6	
Europe	8	1	1
Central South America		3 -	
Industry Employed			
Construction	5	2	1
Healthcare	2 (social worker)	2 (nurses)	
Hospitality	3	2	1
Manufacturing	2	1	
Retail	6	7	1
Oil and Gas	5	1	
IT/other		1 (IT)	

This study included a diverse group of participants: 23 (55%) men and 19 (45%) women between 18 and 55 years old. The participants, who were either permanent residents or citizens, hailed from a variety of regions, including Asia (43%), Africa (26%), Eastern Europe (24%), and Central South America (7%). The sample included overseas qualified engineers, lawyers, business managers, health care professionals (doctors, social workers, nurses), and IT professionals. At the time of the interview, nine participants who had sustained injuries were not working. Although the recruitment process did not specifically target qualified recent immigrants, thirty-four (81%) of the participants had undergraduate (38%) or graduate degrees(38%) or specialized trades qualifications from overseas (24%). They were engaged in skilled work in their country of origin. Currently, they are doing jobs for which they are overqualified. An immigrant is considered overqualified if the skill requirement of their current job in Canada is lower than their educational attainment and lower than the skill requirement of their job before they migrated to Canada or lower than the skill requirement of their job in Canada (44).

At the time of the interviews, they worked in retail (33%), construction (19%), the gas and oil sectors (14%), hospitality (14%, cooks, cleaners) and health care (10%). Others were in manufacturing (warehouse workers, shop and machine assistants) and information technology. Thus, a significant difference between this immigrant group of employees and other precarious workers is that almost two-thirds have done more skilled work before their migration and are qualified to do so. Except for the participants who worked in health care and the oil and gas industry, none had formal work contracts and were employed casually or temporarily. Only the nurses and social workers had successfully found work in their fields, though much below their experience level. All the participants perceived that their jobs were unsafe.

Twenty-four (57%) participants had sustained and reported a physical workplace injury/illness, and three participants failed to report their injuries. Fifteen participants (36%) were not injured or did not report any injury. The injuries sustained by the participants varied in intensity and severity. They included back, shoulder, and arm injuries, strain injuries, burns, and psychological injuries arising from experiences like bullying and harassment (experienced by ten), though these latter injuries were not reported.

### 3.2 Mental health issues faced by participants

Most of the participants identified several workplace conditions that created ongoing psychological distress. They described their distress in lay terms like stress, fear, sadness, worry, anger, anxiety, frustration, dissatisfaction/unhappiness, crying, blaming self, not good enough (self-doubt), lacking confidence, shame, embarrassment, inferiority, and guilt. They also reported headaches, frequent body aches and pains, sleep difficulties, and exhaustion. Only two participants used the terms depression and anxiety to describe their mental health condition.

**Figure 1:**
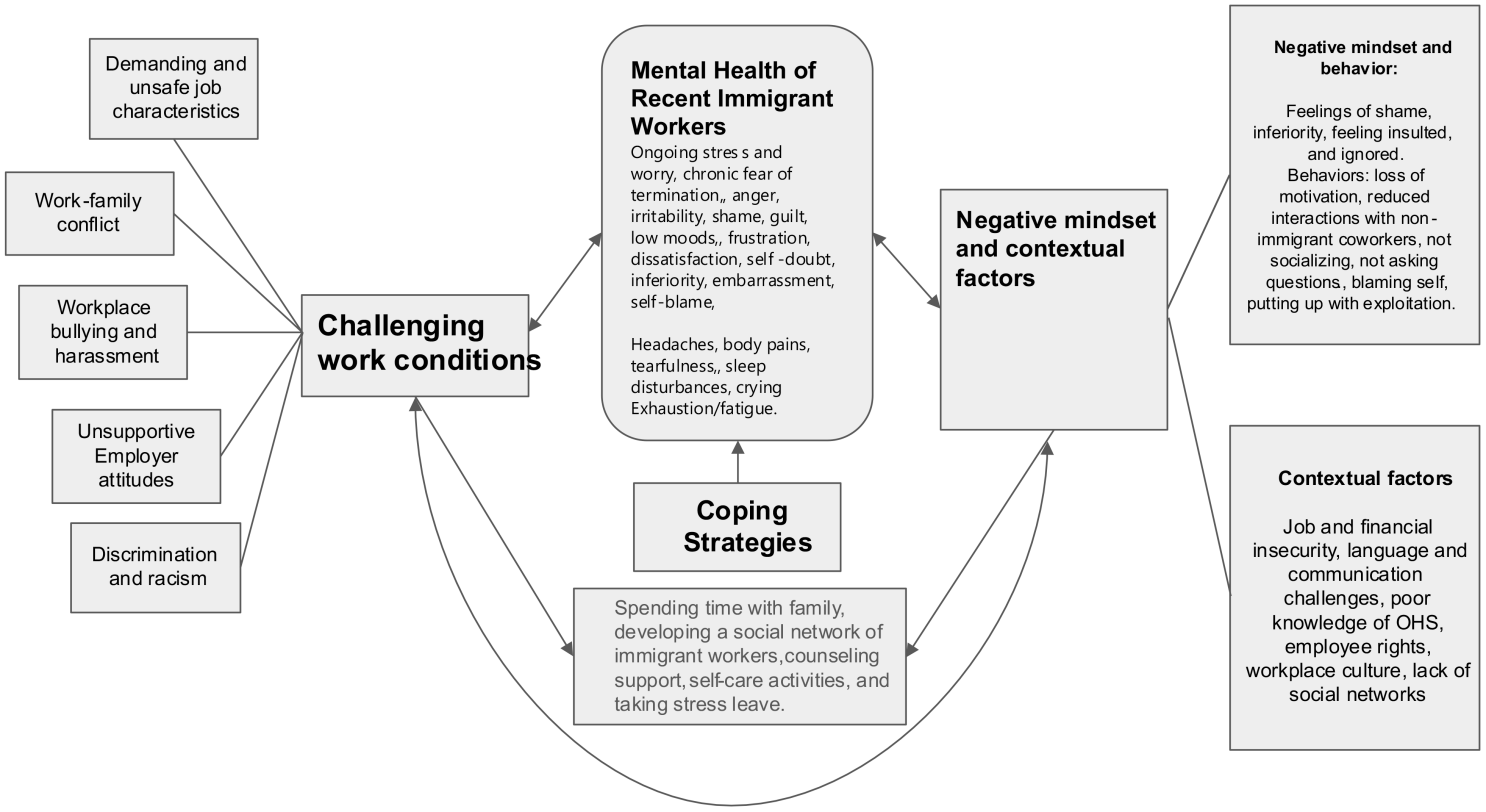
Pathways between mental health, precarious employment, and factors associated with recent immigrant status.

**Figure 1** provides a conceptual framework for the findings. It identifies the four key themes that influenced the participants’ mental health. The first theme, challenging workplace conditions, highlights several workplace factors (sub-themes) that influenced the participants’ mental health. The second theme identifies the mindsets participants developed because of their workplace experiences; the third theme highlights contextual factors related to their recent immigrant status that affected their mental health. The fourth theme, coping strategies, reveals various methods that the participants used to deal with the mental health challenges. The following sections are structured around each theme and highlight how their work conditions affected participants’ health.

### 3.3 Challenging workplace conditions

#### 3.3.1 Job characteristics

Several participants recounted working in adverse environmental conditions on a daily or regular basis and the toll these had on their physical and mental health. These conditions included exposure to toxic chemicals, working in confined spaces, working alone at night, and working outdoors in cold or inclement weather, often leading to accidents like slipping on ice. The impact of becoming physically unwell due to these exposures usually made them dislike going to work. Still, they reported that they could not quit because of the fear of not getting another job and financial insecurity.

*Yes, my job is highly intense. I have to sit down all day. I feel pain in my shoulders and my neck. I have to keep one position repeatedly all day. Sometimes, fumes cause headaches because the smell is very strong. But what can we do? It is part of our job. (E)*

Participants also faced additional stress from the physical demands of the job, which included repetitive work, heavy lifting, and prolonged standing, which resulted in sprain injuries and body aches. Those who worked with dangerous machinery without adequate training and personal protective equipment (PPE) reported being constantly worried that their health may be affected at some point.

Some participants could deduce that their injuries resulted from a mismatch between their high educational qualifications and the jobs that they were currently doing. One of these participants, who had specialized in public health and had worked as a public health specialist back in his country and had no experience with physical labor jobs, shared his mental distress and physical agony because of a spinal disk injury he had sustained in Canada due the heavy lifting he had to do as part of his current job. He attributed his injury to a lack of training and experience in doing labor jobs. Similarly, an overseas qualified medical doctor who had sustained a severe arm injury while working in a low-skilled job in the hospitality industry linked her injury to a lack of experience in doing labor-intensive jobs and shared her grief of not being able to work anymore as a doctor because of being in constant pain.

Participants also shared sleep difficulties arising from working 12-hour shift rotations, making them tired, irritable, and anxious. In addition to the constant worry that she could “kill someone” if she made a mistake, a nurse participant reported feeling “very stressed” because her unit was understaffed. She said extremely busy nurses take shortcuts and sometimes do not follow safety protocols, which leads to injuries. Several participants noted that heavy workload and the pressure to work faster affected their sleep patterns and increased the risk of accidents. A woman participant shared that her blood pressure had shot up because of a heavy caseload and lack of sleep. Five women participants expressed that the physically demanding nature of their work during their pregnancy impacted their health. In one case, the physical and mental strain at work during her pregnancy caused her blood pressure to rise and induced premature labor.

#### 3.3.2 Work-family conflict

At least ten participants spoke about not being informed in advance about their work schedule and asked to work extra hours on short notice. While some were angry for not being paid overtime, others were upset because they had to be constantly available, which created difficulties when planning for other areas of their lives, especially time with family. They reported that the fear of being replaced by other workers (job insecurity) was a significant factor that made them agree to always be available for their employer.

*Sometimes, I have days off, and they call me. I am supposed to go somewhere with my family, and then they call me. ‘We need you! We cannot have a job done unless we have people like you. You have the right to refuse, but when you refuse, your manager will not be happy. I sacrifice and I go to work and then my family would be angry! (AK)*

Women participants who were asked to do frequent evening shifts were very unhappy and often experienced guilt because the shifts came in the way of being available for their families, especially dependent children. A woman participant who frequently did evening shifts and had two jobs explained:

*My son used to come back home after school around 4 pm but at that time I had to be in the workplace. I could not take care of my child, could not feed him, or could not stay with him. After coming home at 10 PM, I often found that my son was sleeping without eating his dinner. My son was also deprived of my care when I had to work very early in the morning. Sometimes, it happened that I could not see my son all day. (U)*

In addition, participants who were experiencing physical health problems and mental stress at work said that they could not share some of their issues with family members. One of them expressed:

*When I am sick, the entire family is affected … After coming from work, I cannot say I feel pain in my leg. I need to cook food for my family, and I have to take care of my son. So, if I do so, it would create further mental stress for all of us … That’s why I did not let my family know that I was suffering. (UZ)*

Work demands also affected participants’ ability to make time for self-care. A participant who had been advised to see a medical specialist experienced difficulties making appointments because she worked on the days the specialist was available. While some participants expressed anger at being unable to achieve a work-life balance, others felt guilty because the lack of this balance affected their health and family life.

#### 3.3.3 Workplace bullying and harassment

Ten participants reported experiencing harassment and bullying from their supervisors and coworkers. They said they were unaware these were occupational health and safety issues and could be reported. They also expressed worries about reprisal if they reported. In some cases, the perpetrators were immigrant supervisors and coworkers. A participant who had developed anxiety and sleeplessness and was taking antidepressants due to bullying and harassment by her team members who were from a different ethnic group described enduring several weeks of mental agony before she could muster the courage to inform her senior supervisor of her desire to quit.

At least 25% of the participants described their work environment as toxic, with minimal trust between immigrant and Canadian-born employees. One of them said that name calling (for example, ‘curry for South Asian) is a common form of harassment. He reported that while some Canadian-born coworkers viewed using such names as playful teasing, many new immigrants experienced this as a form of harassment that they had to put up with to be perceived as good team members. Additionally, the majority of participants reported that many new immigrants find it difficult to report harassment due to language barriers and a lack of social connections.

*Because we have language barriers, we do not know many people here; we do not know where to go if we have any problem in our workplace … We do not know how we should tell our problems to others. That’s why we cannot tell our stories to others (coworkers and supervisors), and we try to refrain from complaining about others when they are very rude to us (CZ).*

#### 3.3.4 Unsupportive employer attitudes

For most participants, their employers’ attitudes towards them were the primary source of workplace stress. At least 50% of the participants said they felt financially and emotionally exploited by their employers/supervisors. Some employers did not pay overtime, while others did not provide adequate lunch breaks. A participant was told he could take breaks only when the “workplace was not busy,” which rarely happened.

*Usually, I work 40 hours per week. If I work more than 44 hours, I’m supposed to get overtime pay, but it never happens. I have never received any overtime pay from my company even when I work 50 hours per week because we have to catch up on the schedule. I don’t want to lose my job. So, I just keep silent. (F).*

Many employers also set unrealistic expectations and targets that did not give participants a sense of control over their jobs and made them feel devalued. A participant who was employed as a team supervisor and had recently quit her job said:

*I was asked to fulfill what felt like impossible targets. So, it was an environment where it was impossible to succeed. I wanted to be a good boss for my employees, and I felt I didn’t know how to do that anymore. So, it definitely had a direct impact on my mental health and impacted my ability to sleep and have emotional regulation. It’s just a very hostile and toxic space to be in. (B)*

In some workplaces, the benchmark for work performance was set high to match that of the fastest workers, inadvertently forcing all workers (and not just immigrant workers) into unhealthy competition. This unhealthy competition reinforced participants’ beliefs that their employers valued productivity and profit more than their physical and mental safety. Participants who were caught in this situation and could not keep up with the pace of work feared they would make a mistake and be laid off.

Unsafe and poor working conditions added to the fear and anxiety participants experienced. Several participants expressed that their employers did not enforce safety compliance and escaped punishment because of government safety departments’ lack of safety inspections. Worse still, refusing unsafe work was not an option for these participants. They knew of other immigrant workers who were fired for speaking up or refusing hazardous work. The constant threat of termination and financial insecurity led to further mental health decline, as illustrated by the following quote from a participant:

*Employers, immigrant or Canadian, always keep immigrant workers under pressure. They know that these workers need a job to survive here, and they do not have that many options, like the Canadian-born people, to get another job. We do not know many people, we don’t know many rules and regulations, so we do not dare to complain. We have to struggle in the workplace, and we have to bear many different types of stress and pain day after day because we need the job! (T)*

Compounding this stress, employers provided little or no information to injured participants on what post-injury support is available and how to access it. Their supervisors would often try to shrug off responsibility by blaming the participant for the injury, not advising them to put in a worker’s compensation claim, not providing workplace accommodations after the injury, or not offering a gradual return-to-work plan commensurate with the worker’s pace of recovery. Lacking support from their employers, many injured participants felt disenfranchised and had to learn how to navigate the complex worker’s compensation system. For injured workers who were already under financial stress because of income reductions, their anxiety was further exacerbated as they were left wondering if there would be a job for them to return to.

#### 3.3.5 Discrimination and racism

The majority who reported racism experienced this from white supervisors or coworkers, though a small number reported experiencing racism from immigrant supervisors and coworkers. Participants who had experienced racial discrimination from white supervisors reported feelings of anger and demotivation when they noticed that their white Canadian peers were treated more favorably, were held to a lower standard of accountability, and were not written up for being tardy or for missing work. One of these participants shared his experience.

*We have these guys (coworkers), they are White. When they come to work you can see the kind of relationship, she [supervisor] has with them because she is also White and they will talk, but once we show up, she will just direct you to go down there and work. She will never check up on you to see how everything is going. They (white coworkers) could be texting on their phones, and she would not say anything, and we (immigrants) were not even allowed to use our phones (I).*

Participants expressed feelings of hurt and disappointment when they were overlooked for promotions despite their hard work or when their supervisors did not value their qualifications and experience because they were from “poor” (a participant’s own words) countries. A skilled and experienced welder participant recounted how he felt treated like “garbage” despite his specialist skills and was told to be a welder’s assistant. Some participants experienced discrimination because of their poor language skills, while others experienced discrimination because of their religion.

*Discrimination is everywhere. Especially if a person has poor English. At my first job, this language discrimination almost killed me. I felt depressed. Without proper English, you are nobody (V)*

*As I see in my workplace, most of the parents do not like a childcare worker wearing hijab. That’s why you will not see many Muslim women with hijab in my workplace. There are many parents who feel a lack of safety for their children who are under the care of a hijabi woman (U)…*

Many participants expressed dissatisfaction, noting a trend where immigrant employees faced a higher likelihood of job terminations or layoffs, often within their initial 3-month probationary period. They attributed this to a lack of awareness among new immigrants about occupational safety and failure to comply with workplace safety regulations. Additionally, new immigrant employees were told to take up jobs that Canadian-born employees did not like, and they felt that their performance was over-scrutinized. Others felt supervisors and coworkers perceived them as less capable. They constantly feared making mistakes and had the added stress of demonstrating competence. In most cases, however, participants tolerated racism, discrimination, and exploitation and rarely complained because of job insecurity and financial instability.

### 3.4 Developing a negative mindset

Several participants expressed feeling embarrassed due to their language barriers and accents. Some acknowledged feelings of inferiority stemming from a lack of familiarity with Canadian culture and an inability to “speak fast” like their Canadian-born peers. They felt offended when coworkers used terms that they did not understand towards them. Those who were used to the more friendly and informal working environments in their country of origin (for example, greeting coworkers, sharing food, and sharing information about family) felt ignored and ostracized when coworkers and supervisors did not acknowledge their greeting. One commented, “This kind of situation makes me upset and frustrated, and I lose interest in doing my work.” Another participant related how she felt insulted each time she asked questions, and her supervisor responded with irritation. Such experiences made some participants develop negative mindsets and behaviors. They experienced loss of motivation and interest in their work. They started limiting their interactions and socialization with their white Canadian coworkers. They kept to themselves and, as reported by one worker, “minded my own business.” Self-consciousness about their accent, lack of confidence in their language skills, and fear of being insulted restricted them from asking questions, an essential aspect of Canadian workplace norms. There were participants who, despite working in unsafe conditions, blamed themselves for their injury, for either being careless or forgetting what was taught during their orientation regarding safety procedures at work. Referring to some of her newly hired immigrant colleagues who had sustained injury and were blaming themselves, a participant explained:

*By blaming themselves, these employees are normalizing injustice. They are very stressed and frustrated but accept exploitation because they have mortgages on their house and loans to repay (L).*

### 3.5 Contextual factors

While job and financial insecurity were the critical contextual factors that made participants put up with distressing workplace conditions, language and communication challenges added to their distress. Other contextual problems that caused frustration included inadequate occupational health and safety (OHS) knowledge, poor understanding of Canadian workplace culture, and lack of social networks. Regarding OHS, most participants knew they should follow safety rules and use protective gear. Still, their knowledge of OHS was insufficient, especially concerning their employee rights. While many participants knew they could refuse unsafe work, they were unaware of the employer’s responsibility to provide OHS training and a safe workplace. They were not informed about the procedures to report injury and the availability of support like worker’s compensation and disability leave. This resulted in many injured workers experiencing considerable stress after sustaining injury. Very new participants, for example, those under five years of residency in Canada, did not know they could have short breaks between work hours and were entitled to overtime pay. Lack of awareness made these participants take on unsafe work and agree to work longer hours, sometimes without overtime pay. Reflecting on the behaviors of some of his immigrant coworkers, one participant commented:

*They keep silent because they want to prove themselves to the employer that they can do everything better and fast. Sometimes they don’t see the shortcut they take can affect their job quality and they will end up with something unsafe (A).*

Another contextual factor that added to participants’ feelings of job insecurity and frustration was the lack of social networks, like relatives and friends whom they could turn to for advice on Canadian workplace cultural norms and safety legislation. Referring to these limitations, a participant explained:

*For Canadians, they might have their family members, like relatives or parents to provide information (about workplace norms, safety legislation, and worker rights) to them. They may have already accumulated different experiences or knowledge such as workplace safety and working norms from Canadian society. However, for immigrants, they do not have the people who could provide this information. It is very difficult for them to obtain the same kind of information. It could become the excuse for their termination (C).*

### 3.6 Coping with distress

Participants showed resourcefulness in coping with workplace-related stress, utilizing diverse individual, cultural, and contextual strategies. These can be grouped into three categories: distracting and distancing, changing mindset and developing positive thinking, and actively seeking friends and community support.

#### 3.6.1 Distancing and distracting

Participants tried to distract themselves from their work-related stress by participating in activities like attending church, praying, watching movies with children, window shopping, pursuing hobbies, or engaging in housekeeping. Others undertook self-care activities like yoga, salsa dancing, walking, biking, or gym. At least two participants had distanced themselves from work by taking stress leave. However, taking leave did not help as the conditions at work did not change. This led to these participants eventually quitting their jobs. A third participant mustered the courage to speak with her senior supervisor about the harassment she was experiencing and was able to get a transfer to another department.

#### 3.6.2 Changing mindset and developing positive thinking

A participant who felt isolated in her workplace because of language barriers started attending English courses to improve her language skills. Unfortunately, she had to quit because, in her own words, *“It was almost a waste of time. It was very difficult to study anything after 10 – 12 hours of the working day. My brain did not want to accept new information”.* Another participant said he was trying to become more friendly with his Canadian-born co-workers and not take their teasing personally. Volunteering at her child’s school helped a participant learn more about Canadian culture, which, in turn, was helpful at her workplace. A participant who was very unhappy at his job said his mindset changed after going to church and participating in some community events. He started to feel that he was fortunate that he could immigrate to Canada and find a job, although the job that he had was not the best.

#### 3.6.3 Seeking friends and community support

Strategies under this category included seeking friends with similar experiences and developing a social network comprising immigrants from their own country and other countries. These underscore the significant role of community support to help immigrants cope with work-related stress. Only one participant said that he was seeking the help of a counselor to help him deal with work stress. Two participants were being treated by their doctors for depression and were on medications. None of the other participants reported seeking professional help to deal with their stress.

## 4 Discussion

This qualitative study examined the work experiences of recent immigrants who were engaged in precarious employment and the influence of these on their mental health. The study findings add to the small but growing body of evidence (7, 11–14, 22, 24, 45) that workplace challenges like those experienced by the study participants, job insecurity, and financial instability can deleteriously impact the mental health of precariously employed workers. **Figure 1** offers a nuanced understanding of the complex intersections of mental health, unsafe employment conditions, negative mindsets that employees may develop, contextual factors, and coping strategies that employees may use to deal with their challenges. While the hazardous work conditions and risks identified in this study can adversely affect the mental health of all employees engaged in precarious work, in the case of recent immigrants, these intersections can compound the stressors impacting their mental health because of the multiplier effect of contextual factors associated with their immigrant status.

The psychological problems identified by the study participants reflect a predominantly psychosocial rather than a clinical conceptualization of mental health and well-being. As indicated earlier, mental health assessment in this study was based on subjective accounts of participants’ mental state. Given the personal nature of all psychological experiences and ongoing debates about the utility of diagnostic measures (39), this was not considered a limitation. Since no standard mental health measures were used, it was difficult to say from the participants’ reports whether they were experiencing distress or illness (depression, anxiety, or common mental disorders) (46, 47). Participants’ reports of body aches and pains, crying, headaches, exhaustion, and difficulties with sleep provide valuable insight into how mental ill health (distress/illness) may be expressed in immigrant populations (46, 47), a finding that is significant for service providers and employers.

With specific reference to the eighty-one percent of study participants who were highly skilled but performing jobs much below their qualifications and skill level, current findings suggest that such immigrants may experience a higher risk of injury and mental health problems compared to other employees in precarious employment. These findings complement previous research (48). Overqualified participants in the current study knew that their injury was due to a lack of fit between their qualifications and current job demands and their work not matching their interests. One of the reasons for their injury, as indicated by current findings, is that many participants had no experience performing labor-intensive jobs in their country of origin. Not having received any training to perform such jobs in Canada, their bodies may not be able to adapt quickly enough to manual labor demands as it may take 8- 12 weeks for muscles to adapt, and between manual labor, rest is needed for the tissues to heal (49, 50). This can partly explain their physical distress/injury. Second, it has been suggested that many qualified and skilled immigrants derive their sense of identity and self-perception from their social and professional status (19, 51). Being granted PR status based on their professional abilities and skills, the failure to get professional jobs, and the loss of identity and social status accompanying this can lead to unhappiness, frustration, and anxiety (19, 51). Additionally, overqualification can lead to de-skilling (20). This process refers to the loss of skills and knowledge that accompanies the underutilization of immigrants’ skills, an essential factor that can impact mental health (52). In summary, the impact of precarious employment conditions on overqualified recent immigrants may be more profound than on other precariously employed persons due to their higher risk of sustaining an injury and factors such as deskilling, loss of identity, and social and professional status.

Understanding the negative mindsets of recent immigrant employees is complex, as multiple factors can influence these. There is little research that has examined how negative mindsets develop and the impact of these on the employee’s attitudes and behaviors. Current findings suggest pathways through which these may develop in new immigrant employees and affect workplace behaviors. As indicated by Wallace et al. (53), these behaviors could be emotional distress responses of participants as a reaction to experiences of racism and discrimination. Recent immigrants may use these responses to protect themselves from future hurt. However, these responses can be interpreted negatively by employers/supervisors, leading to recent immigrant employees being perceived as less intelligent, trustworthy, and employable and failing to integrate (54, 55). Such perceptions can influence the employer’s interactions with these employees, which, in turn, can negatively impact them. The urgency and importance of addressing the negative mindsets of immigrant employees is evident. It requires a multidimensional approach that combines supportive policies, cultural awareness training, mental health interventions, and efforts to foster inclusive work environments.

Coping strategies can play a significant role in reducing the impact of stressors. In terms of the coping strategies used by participants, many of these align with previous studies (56). The findings suggest that the participants used constructive and nonconstructive coping strategies. Nonconstructive strategies, such as taking stress leave, did not help their situation as their work conditions did not change. In contrast, constructive strategies were helpful, like developing a social network or changing their mindset by reinterpreting their work and life situation as a positive experience. The failure on the part of participants to use professional help to cope with their mental health issues could be due to their lack of knowledge and familiarity with available services, cultural taboos about using these services, and language barriers. Further research on the effectiveness and accessibility of coping strategies is necessary to improve the well-being of immigrant worker populations in the workplace.

The findings of our study, while focused on recent immigrants, have significant implications for the mental health and well-being of all precariously employed individuals. Many of these individuals perform jobs that are below their capacity, qualifications, and skill levels (7). Given that poor mental health can have a substantial impact on productivity, increase the risk of accidents, and affect various aspects of one’s life, policymakers should urgently address the growing prevalence of precarious employment and its impact on employees. The following section discusses the policy and practice implications for recent immigrants.

### 4.1 Implications for policy and practice

The government, service providers, and employers have a significant role in reducing the health impact of precarious employment on recent immigrants who are at greater risk than other employees of experiencing underutilization of their qualifications and skills and sustaining a physical and psychological injury because of the contextual factors associated with their immigrant status. Although detailed policy and practice recommendations are beyond the scope of this paper, we provide some practical suggestions based on research and current findings. We recommend that Canada’s current policy of inviting skilled immigrants into the country but not ensuring they get suitable employment be reviewed to ensure that immigrants can utilize their skills and qualifications in a just and inclusive work environment. The current policy violates social and occupational justice principles by failing to provide equitable opportunities for meaningful and appropriate employment. We also recommend that the rhetoric of Canadian work experience promoted by the state to facilitate labor market integration of new immigrants be set aside. To demonstrate Canadian work experience, immigrants must resort to unpaid or low-paying jobs, and some may even sustain work injuries in the process, as in the case of some participants in the current study. These practices result in the deskilling of immigrants and subject them to “processes that can reproduce immigrant workers in the lower echelons of a gendered and racialized labor market” [(57), page 3]. Occupational therapists (OTs) can play a crucial role in addressing occupational justice for new immigrant workers. By advocating for fair and inclusive employment practices, OTs can help ensure that immigrants have access to work that aligns with their skills and qualifications. Furthermore, OTs can develop targeted interventions to support immigrants in overcoming barriers to equitable employment, thereby promoting their overall well-being and integration into the labor market.

To minimize the risk of work injury among recent immigrant employees, the government should invest in providing OHS training before they take up employment or soon after. The government can fund Immigrant-serving organizations to offer this training to new immigrants. Equipping immigrants with OHS knowledge is a crucial first step, as this will help them understand their rights and obligations, build their confidence and empower them to navigate the workforce, reduce vulnerability to workplace injury, and improve mental health. To minimize the risks of immigrant skill underutilization, it has been suggested that the government should introduce guidelines for bridging immigrants’ foreign credentials and working experience with their settlement and integration (58). It can invest in practical workforce development and training services for immigrants who want to access or transition into safer employment. The government can invest in occupational therapy services through employment services and immigrant-serving organizations, including the following: job matching and career counselling to advise and assist newcomers in accessing specific professional education and competencies, language training, fast-track programs, and fee waivers from educational institutions (59); cultural awareness training which can include ‘soft’ skills training for improving workplace communication and relationship building, teaching new immigrants about Canadian workplace culture and norms, culturally appropriate ways of interacting with coworkers and supervisors, confronting racism, workplace bullying, and harassment (52); and mentorship programs for personal and emotional support, workplace knowledge and professional network development (60). Such programs have the potential to mitigate the risks of deskilling and underutilization of immigrant skills and the development of negative mindsets and their associated health impacts.

Employers have a critical role in improving workplace conditions for all employees engaged in PE, specifically for vulnerable workers like qualified recent immigrants. However, given the steady rise in precarious employment, which has encouraged many employers to maintain a flexible workforce and compromise on employment and safety standards (4), and growing evidence of the adverse impact of PE on employee health, the government has the responsibility for instituting measures that can increase employer accountability towards employees and create safe and inclusive workplaces that can promote the mental health and well-being of all employees, especially those who are most at risk like skilled newcomers. Though research on workplace interventions for reducing the impact of PE on employee health is in its early stages (61, 62), we provide the following practical suggestions based on participants’ experiences. The government can (a) support the development and dissemination of education for employers on the health impacts of PE on employees, especially new immigrants, and the work conditions and contexts that give rise to these; (b) implement stricter laws and regulations that can serve as incentives to employers to create safe and culturally inclusive workplaces (61, 63), and (c) support the development and uptake of cultural diversity training for employers (HR personnel and managers) that can help to address their biases towards new immigrant employees, their overseas qualifications and skills (6). Practical strategies that employers, on their part, can undertake include implementing policies that offer more predictability in hours, which can counter responses of overwork and presenteeism (40) and address discrimination and harassment in the workplace. Employers can provide mentors to new immigrant employees so that they can integrate and have equal opportunities for career advancement within the organization. Employers (and human resource managers) can invite healthcare providers to provide training that will help managers recognize the signs of mental ill health, especially among their immigrant employees, the workplace challenges and contexts that give rise to these, and the cultural taboos that are associated with talking about mental ill health. This can help in the early recognition and referral of troubled employees to healthcare providers for support. In addition to these measures, occupational therapists can offer concrete support by developing workplace mental wellness programs, tailored specifically for immigrant employees. They can conduct workplace assessments to identify and mitigate potential physical and psychological hazards that disproportionately affect immigrant workers. By collaborating with employers, OTs can help design culturally sensitive work environments and provide ongoing support to ensure the well-being and productivity of immigrant employees.

The uptake of the suggested recommendations depends on current neoliberal governments’ willingness to invest in the workforce development of newcomers and not leave these solely on the shoulders of new immigrant employees or their employers. It also depends on employers’ interests and resources to create safe and inclusive workplaces. Delays in implementation will not only increase the risk of poor mental health and disability among vulnerable workers but can affect the country’s economic and social development.

### 4.2 Study limitations

The following are some of the study limitations. First, the sample for this study was drawn from only two geographic sites in a province. Since both sites were urban, we do not know about the challenges that recent immigrants have faced in rural and semi-rural areas. Second, the study sample is weighted toward professionally qualified immigrants, though the recruitment strategy didn’t specifically target this group. Third, the sample was primarily comprised of service users of immigrant-serving organizations. These service users’ experiences and health effects may differ from those of recent immigrants who do not use these services. Future research must include service users from this latter group. Fourth, this study focused only on recent permanent residents. Future research should include a comparison with other vulnerable populations, such as temporary foreign workers, which can provide information on whether employees with PR status in PE are better off than other immigrant groups. Fifth, given this study’s design, it was not possible to determine the role of external factors like migration and loss of family and friends and their influence on participants’ mental health. Despite these study limitations, the research results are nonetheless significant regarding their implications and contributions for Canada and beyond.

## 5 Conclusion

This study is one of the first few qualitative studies to examine the mental health challenges of recent immigrants in precarious employment contexts. More than two-thirds of the study participants were overqualified for their jobs. The study findings extend beyond workplace conditions and offer a nuanced depiction of the various factors influencing mental health. The findings highlight several psychological problems that participants experienced and four intersecting key themes that influenced their mental health. These include challenging workplace conditions, recent immigrant mindsets, contextual factors, and coping strategies used to deal with distress. The study findings suggest that recent immigrants taking up precarious employment are at high risk of developing mental health problems and that overqualified immigrants may be more vulnerable to sustaining both physical and psychological health problems. Based on the findings, a multidimensional approach is proposed for addressing workplace conditions and promoting the mental health of recent immigrants. This approach underscores the urgent need for policy and legislation changes, as well as programs for creating awareness among employers about the importance of the mental health of recent immigrant employees and the crucial role of employers in creating safe and culturally friendly workplaces where each worker feels safe, respected, and valued. The approach also recommends that recent immigrant employees receive occupational health and safety training, access services to learn about Canadian workplace norms and culturally appropriate ways of interacting with coworkers and supervisors and receive timely health care support.

## Data Availability

The original contributions presented in the study are included in the article/supplementary material. Further inquiries can be directed to the corresponding author.

## Appendix

Appendix: Guiding Questions

– Information about self.

– Description of their job

– Kind of experience, training, or educational qualification received

– How do they understand occupational/work safety and workers’ rights?

– About awareness of the right to refuse work.

– How do safety issues in the workplace affect health, mental health, and well-being?

– Satisfaction with workplace conditions and relationships?

– How does work impact family life and vice versa?

– How they have kept themselves from not being injured

– Reasons why immigrant workers refrain from reporting workplace injury/illness or claiming worker’s compensation for injury

– Familiarity with the workplace’s hiring and termination practices?

– Awareness of job benefits

– How can occupational safety and well-being be improved?

– Perspectives on overall well–being:-

Additional questions for injured

– Experience of injury, type, severity

– How do they cope with circumstances of post-injury/illness?

– How do they cope with their financial situation after an injury?

– Awareness of the company’s policies and practices if anyone sustains a workplace-related injury.

– Awareness of support after a work injury, access to support, barriers to accessing support, and satisfaction with support received.

– Types of workplace accommodation provided

– Views on improving your return-to-work outcomes?
